# mSphere of Influence: Compartmentalized cAMP signals in American trypanosomes

**DOI:** 10.1128/msphere.00635-23

**Published:** 2024-02-05

**Authors:** Noelia Lander

**Affiliations:** 1 Department of Biological Sciences, University of Cincinnati, Cincinnati, Ohio, USA; California State University Fullerton, Fullerton, California, USA

**Keywords:** adenylate cyclase, cyclic AMP, differentiation, environmental sensing, phosphodiesterases, signaling domains, *Trypanosoma cruzi*

## Abstract

Noelia Lander works on cell signaling in American trypanosomes and studies the role of cyclic adenosine monophosphate (cAMP) microdomains in environmental sensing and differentiation. In this mSphere of Influence, Dr. Lander reflects on three research articles in different eukaryotic models that had impacted on the way she thinks about the regulation of cAMP signals in *Trypanosoma cruzi*, the etiologic agent of Chagas disease. The articles “FRET biosensor uncovers cAMP nano-domains at β-adrenergic targets that dictate precise tuning of cardiac contractility” (N. C. Surdo, M. Berrera, A. Koschinski, M. Brescia, et al., Nat Commun 8:15031, 2017, https://doi.org/10.1038/ncomms15031), “Cyclic AMP signaling and glucose metabolism mediate pH taxis by African trypanosomes” (S. Shaw, S. Knüsel, D. Abbühl, A. Naguleswaran, et al., Nat Commun 13:603, 2022, https://doi.org/10.1038/s41467-022-28293-w), and “Encystation stimuli sensing is mediated by adenylate cyclase AC2-dependent cAMP signaling in *Giardia*” (H. W. Shih, G. C. M. Alas, and A. R. Paredez, Nat Commun 14:7245, 2023, https://doi.org/10.1038/s41467-023-43028-1) influenced her current hypothesis that cAMP signals are generated in response to environmental cues leading to changes in membrane fluidity at the flagellar tip and the contractile vacuole complex of *T. cruzi*, structures where cAMP mediates key cellular processes for developmental progression.

## COMMENTARY

Many unicellular eukaryotes, pathogenic and free-living alike, transition between developmental forms during their life cycle. How these organisms sense the environment to determine when and where to differentiate is largely unknown. Cyclic adenosine monophosphate (cAMP) is a universal second messenger that mediates a variety of cellular processes in eukaryotic cells, including differentiation. After several years in quiescence, the study of cAMP signaling in trypanosomatids has become a hot topic again, as evidenced by recent publications in the field (1–6). I was drawn to this subject from the beginning of my independent research on signal transduction pathways in *Trypanosoma cruzi*, the agent causing Chagas disease. Understanding the role of cAMP in environmental sensing could lead to the development of strategies to interrupt the parasite’s life cycle and control this silent but deadly disease. Our recent work revealed the presence of two putative cAMP microdomains in *T. cruzi*: the flagellar distal domain and the contractile vacuole complex, compartments specialized in cell adhesion and osmoregulation, respectively (4). Studies involving cAMP signaling in three different eukaryotic models have strongly influenced the current direction of my research (1, 7, 8). The first one was performed in cardiomyocytes by the group of Manuela Zaccolo (7), which elucidated the role of three cAMP nanodomains (sarcoplasmic reticulum, plasmalemma, and microfilaments) in generating signals of variable amplitude and kinetics in response to β-adrenergic receptor stimulation. Using a FRET-base cAMP sensor (CUTie), this group elegantly showed that cytosolic cAMP is less involved in regulating cell function than cAMP compartmentalized in nanodomains, where the presence of regulators (phosphodiesterases [PDEs]) and effectors (protein kinase A [PKA]) determines the specificity of the hormonal response. Importantly, this work showed the key role of PDEs in regulating the localization, duration, and amplitude of cAMP signals, which made me reflect on the possible presence of similar domains in *T. cruzi*, as specific PDEs have been found in different subcellular compartments of this parasite (9–13). Overall, the study of Zaccolo’s group (7) challenged the long-standing idea that cAMP is a long-range second messenger synthesized at the plasma membrane that diffuses to activate distant effectors. The new concept of compartmentalized cAMP signals eliciting specific cellular functions has been transformative in the field and could explain the specificity of localized signals in trypanosomes.

Another study that influenced the way I think trypanosomes use cAMP signals to sense their environment was published by the group of Isabel Roditi in 2022 (1). This work provided the first mechanistical evidence that cAMP-mediated pH taxis is used by African trypanosomes as an environmental cue to coordinate their collective movement on semi-solid surfaces, a behavior known as social motility (SoMo). This group found that procyclic forms (PCFs) can generate pH gradients through glucose metabolism and that SoMo patterns in these populations are determined by pH taxis. Then, performing transcriptomic analysis of cells in different regions of the parasite community after exposure to acid or alkali, they identified positive and negative regulators of SoMo involved in cAMP signaling. They concluded that early PCFs are attracted by alkaline pH and identified cAMP response protein 3 (CARP3) as a key player in pH sensing and establishment of infection in the tsetse fly. Notably, pH sensing was completely abolished in the absence of phosphodiesterase B1, a flagellar PDE whose depletion alters cAMP signals generated at the flagellar tip (1, 14). These results support the role of PDEs in regulating compartmentalized cAMP signals in trypanosomatids. Roditi’s group study also challenged the previous hypothesis that trypanosome adenylate cyclases (ACs) act as membrane receptors activated by external ligands directly binding to their extracellular N-terminal region (15, 16). Altogether, these findings led me to hypothesize that microenvironmental cues could activate *T. cruzi* ACs in at least two putative cAMP signaling microdomains: the flagellar distal domain and the contractile vacuole complex. Three cAMP signaling components share dual localization in these microdomains: TcAC1, TcAC2, and TcCARP3, and are involved in cell adhesion, metacyclogenesis, and osmoregulation in *T. cruzi* (4). But there are still key unanswered questions. How are TcACs activated in these two compartments in the absence of G protein-coupled receptors (GPCRs)? What common factor activates these transmembrane proteins in two different cellular structures? A recent study published by the group of Alexander Paredez sheds light on the possible answer (8). In this work, the authors demonstrated that membrane fluidity is a key factor in the regulation of cAMP signals that trigger encystation in *Giardia lamblia*, a parasite that also lacks GPCRs. Using lipid raft markers and a genetically encoded cAMP sensor, this group showed that cholesterol depletion leads to an increase in membrane fluidity elevating intracellular cAMP levels and activating PKA. They identified GlAC2 as the enzyme involved in *Giardia* encystation, a process mediated by cAMP in this parasite. This study reinforced the importance of developing specific sensors to record the generation of cAMP signals in any organism of interest. It also introduced the concept of membrane fluidity as a key regulator of cAMP signals in protozoan parasites.

Together, these studies provided essential evidence to support our current working model where different environmental cues producing changes in membrane fluidity (pH, temperature, osmolarity, ionic composition, cell contact) alter the composition of AC-containing lipid rafts, thus modulating cAMP synthesis. We now have strong evidence supporting the idea that specific PDEs in each subcellular compartment regulate the duration, intensity, and diffusion of cAMP signals, activating effectors near these microdomains. Mounting evidence in protozoans and other cell types support the concept that membrane fluidity alterations could modify the proportion of enzymatically active AC dimers in trypanosomes. While the mechanism of activation of ACs in *T. cruzi* is still elusive, it is clear that cAMP plays a crucial role in environmental sensing and developmental progression. Targeting this signaling pathway could be essential to disrupt the life cycle of *T. cruzi* and control the transmission of Chagas disease.
